# National survey of family physicians to define functional decline in elderly patients with minor trauma

**DOI:** 10.1186/s12875-016-0520-1

**Published:** 2016-08-22

**Authors:** Kasim E. Abdulaziz, Jamie Brehaut, Monica Taljaard, Marcel Émond, Marie-Josée Sirois, Jacques S. Lee, Laura Wilding, Jeffrey J. Perry

**Affiliations:** 1 Department of Epidemiology and Community Medicine, Ottawa Hospital Research Institute, Ottawa, ON Canada; 2 Clinical Epidemiology Program, University of Ottawa, Ottawa, ON Canada; 3 Department of Emergency Medicine, University of Ottawa, Ottawa, ON Canada; 4 Department of Family and Emergency Medicine, Université Laval, Laval, QC Canada; 5 Département de réadaptation, Université Laval, Laval, QC Canada; 6 Unité de recherche en traumatologie-urgence-soins intensifs du Centre de recherche FRQ-S du CHA de Québec, Laval, QC Canada; 7 Department of Emergency Medicine, University of Toronto, Clinical Epidemiology Unit, Sunnybrook Health Sciences Center, Toronto, ON Canada; 8 Epidemiology Program, F6, The Ottawa Hospital, Civic Campus 1053 Carling Avenue, Ottawa, ON K1Y 4E9 Canada

**Keywords:** Elderly, Functional decline, Activities of daily living (ADL), Emergency department, Minor injuries

## Abstract

**Background:**

Failing to assess elderly patients for functional decline at the time around a minor injury may result in adverse health outcomes. This study was conducted to define what constitutes clinically significant functional decline and the sensitivity required for a clinical decision instrument to identify such functional decline after an injury in previously independent elderly patients.

**Methods:**

After a thorough development process, a survey questionnaire was administered to a random sample of 178 family physicians. The surveys were distributed using a modified Dillman technique.

**Results:**

From 143 eligible surveys, we received 67 completed surveys (response rate, 46.9 %). Respondents indicated that a drop of at least 3 points on the 28-point Older Americans Resources and Services (OARS) ADL Scale was considered clinically significant by 90 % of physicians. Ninety percent (90 %) of physicians would be satisfied with a sensitivity of 90 % or more for a clinical decision instrument to detect patients at risk of functional decline at 6 months following an injury. The majority of family physicians do not routinely assess the majority of the tasks on the OARS scale for injured elderly patients.

**Conclusions:**

A high proportion of physicians (90 %) would consider a drop of 3 points on the OARS ADL Scale as significant to define functional decline and would be satisfied with a sensitivity of 90 % for a clinical decision instrument to detect such a decline. Any instrument to identify patients at elevated risk for subsequent decline should consider these outcome measures to be clinically useful.

**Electronic supplementary material:**

The online version of this article (doi:10.1186/s12875-016-0520-1) contains supplementary material, which is available to authorized users.

## Background

Functional decline is a reduction in the ability to manage routine activities of daily living (ADL) necessary for self-care because of a decrement in physical functioning. Functional decline is common and a serious problem in elderly patients [1, 2]. Functional decline is associated with lower quality of life, social isolation, and death [3–12]. It is also an important predictor of a number of outcomes including hospitalization [13, 14], prolonged hospital stay [15], need for home care [15] and repeat emergency department visits [12, 16]. It is a very important predictor of disproportionate use of health services (more than 40 % of public health care expenses) by the elderly population as well as admission to institutions [1, 2, 15–17]. Functional decline in elderly patients has been found to be more predictive of mortality than severity of illness or organ damage [18, 19].

There are a number of assessment scales, such as the Older Americans Resources and Services (OARS) ADL Scale and the Identification of Seniors at Risk (ISAR), but none, to our knowledge, exist to screen for functional decline after a minor injury specifically. It is not currently known how much of a decline and sensitivity is considered clinically significant with practicing family physicians. The objectives were to determine family physicians’ subjective views on what constitutes a clinically significant point drop on the 28-point Older Americans Resources and Services (OARS) ADL Scale to define functional decline 6 months after sustaining a minor trauma, and the required sensitivity for any such clinical instruments.

## Methods

### Study design and participants

We conducted a postal survey using a random sample of 178 family physicians selected from a Medical Directory [20]. Our study was conducted in four stages: 1) key informant, in-person interviews (pre-survey), 2) cognitive interviews (draft survey), 3) pilot testing (final draft survey), and 4) final survey. Dillman’s Tailored Design technique was followed for the design and administration of the survey [3].

### Outcome measures

Functional decline is usually defined and measured by a decrease in ability to perform activities of daily living (ADL) items [15, 21]. ADL tasks have been classified into basic activities of daily living (basic ADL; e.g. feeding oneself) and instrumental activities of daily living (IADL; e.g. operating a telephone) that allow an individual to live independently in a community [22]. We used the OARS ADL Scale to determine a minimum clinically important difference to define functional decline [23]. The OARS ADL Scale is a 14-item questionnaire involving seven basic and seven instrumental activities of daily living (ADL/IADL) items rating the patients on their ability to perform the activities independently [24–27]. A person’s score can range from 0 to 28 with the highest score indicating complete independence in performing the activities of daily living and the lowest score indicating that the patient is unable to perform the activities at all [23, 24].

### Questionnaire development

We developed our survey questionnaire following rigorous development process including key informant and cognitive interviews (Additional file 1). The questionnaire was also pilot tested on a convenience sample of local physicians. The key informant interviews were conducted to establish feasibility of the survey and determine ideal methods on gathering information from the physicians. Cognitive interviews, which involve sitting with respondents and observing how the survey is completed, were conducted to evaluate the comprehensibility, clarity, and face validity of the draft survey. The pilot test of the survey was conducted to identify and fix any potential problems with the survey implementation procedure and the questionnaire.

The final questionnaire consisted of 13 questions, broken down into five sections and was printed on two single-sided pages. Survey materials were translated by an official medical translator into French for the French-speaking physicians.

### Survey administration

The final questionnaire was mailed to the final 174 English and French-speaking family physicians in our sample. Each physician was sent a pre-notification letter a week before mailing the first survey questionnaire. The first survey questionnaire included the coffee card, if applicable. Non-responders were mailed a reminder with a questionnaire every 3 weeks. Canada Post’s Xpresspost courier service was used for the final reminder.

The researchers coordinating this study were located at the Ottawa Hospital Research Institute in Ottawa, Ontario, Canada. This study was approved by the Ottawa Health Science Network Research Ethics Board.

### Data analysis

Descriptive statistics on physician responses were calculated and presented. The gap between physician knowledge and actual practice was presented with bar graphs. The minimal clinically important difference in the OARS ADL score and required sensitivities were presented using frequency distributions and boxplots. The potential for non-response bias was evaluated using characteristics of respondents and non-respondents through Chi-squared tests. Data were analysed using SAS version 9.2 (SAS Institute, Cary, NC).

## Results

From the 178 family physicians initially surveyed, 14 had moved and 20 were ineligible as they were no longer practicing or did not see elderly patients. Our response rate was 46.9 % (67 completed surveys from 143 eligible surveys).

The most common practice location of the family physicians was a group setting (58.2 % of respondents). Our results show that more than 73.0 % of the respondents had been in practice for 10 or more years. More details on demographic information of the respondents are presented in Table 1.

**Table 1:** Distribution of respondent characteristics

Characteristic	# (%) of respondents (*N* = 67)
Sex	
Female	34 (50.7)
Age groups (years)	
< 35	3 (4.5)
35–44	29 (43.3)
45–54	19 (28.4)
≥ 55	16 (23.9)
Years in practice	
< 10	16 (23.9)
10–19	14 (20.9)
≥ 20	35 (52.2)
Years residency training	
< 3	48 (71.6)
3–5	12 (17.9)
> 5–9	3 (4.5)
≥ 10	1 (1.5)
Practice setting	
Solo practice	19 (28.4)
Group practice	39 (58.2)
Hospital	6 (9.0)
Other	3 (4.5)
Number of patients seen/week	
≤ 28	1 (1.5)
29–60	12 (17.9)
61–100	18 (26.9)
> 100	34 (50.7)
Number of elderly patients seen/week	
≤ 20	18 (26.9)
21–30	10 (14.9)
31–50	15 (22.4)
> 50	18 (26.9)

We used two demographics to test for non-response bias: corresponding language and the residing region of Canada. Chi-squared tests showed no non-response bias by the language of the questionnaire (*p*-value: 0.169) as well as by region (*p*-value: 0.478). Table 2 summarizes these findings.

**Table 2:** Characteristics of respondents and non-respondents

Characteristic	Respondents	Non-respondents	*P*-value
	% (*n*)	% (*n*)	
Language of the Questionnaire			0.169
English	82.1 (55)	72.4 (55)	
French	17.9 (12)	27.6 (21)	
Region			0.478
Western Canada^a^	31.3 (21)	31.6 (24)	
Ontario	43.3 (29)	32.9 (25)	
Quebec	17.9 (12)	27.6 (21)	
Eastern Canada^b^	7.5 (5)	7.9 (6)	

^a^British Columbia, Alberta, Saskatchewan, Manitoba, Yukon Territory

^b^New Brunswick, Nova Scotia, Newfoundland

Our results indicate that 90 % of family physicians considered a drop of ≥3 points on the 28-point OARS ADL Scale as significant to define functional decline when considering all 14 ADL/IADL items when patients do not have support at home. The physicians considered a drop of ≥4 points significant when patients have support at home (see Table 3). When only considering the seven basic ADLs, physicians considered a drop of ≥1 points significant when patients do not have support at home and a drop of ≥2 points when patients have support at home as clinically significant.

**Table 3:** Required point drop to define functional decline and required sensitivity for a clinical instrument that would meet or exceed expectations for 90 % of respondents

	Point drop or sensitivity
Minimum point drop in all 14 basic ADL/IADL items^a^	
If patient has NO support at home	3
If patient has support at home	4
Minimum point drop in the seven ADL items^a^	
If patient has NO support at home	1
If patient has support at home	2
Sensitivity^a^, %	90

^a^Meet or exceed expectations for 90 % of respondents

**Fig. 1:**
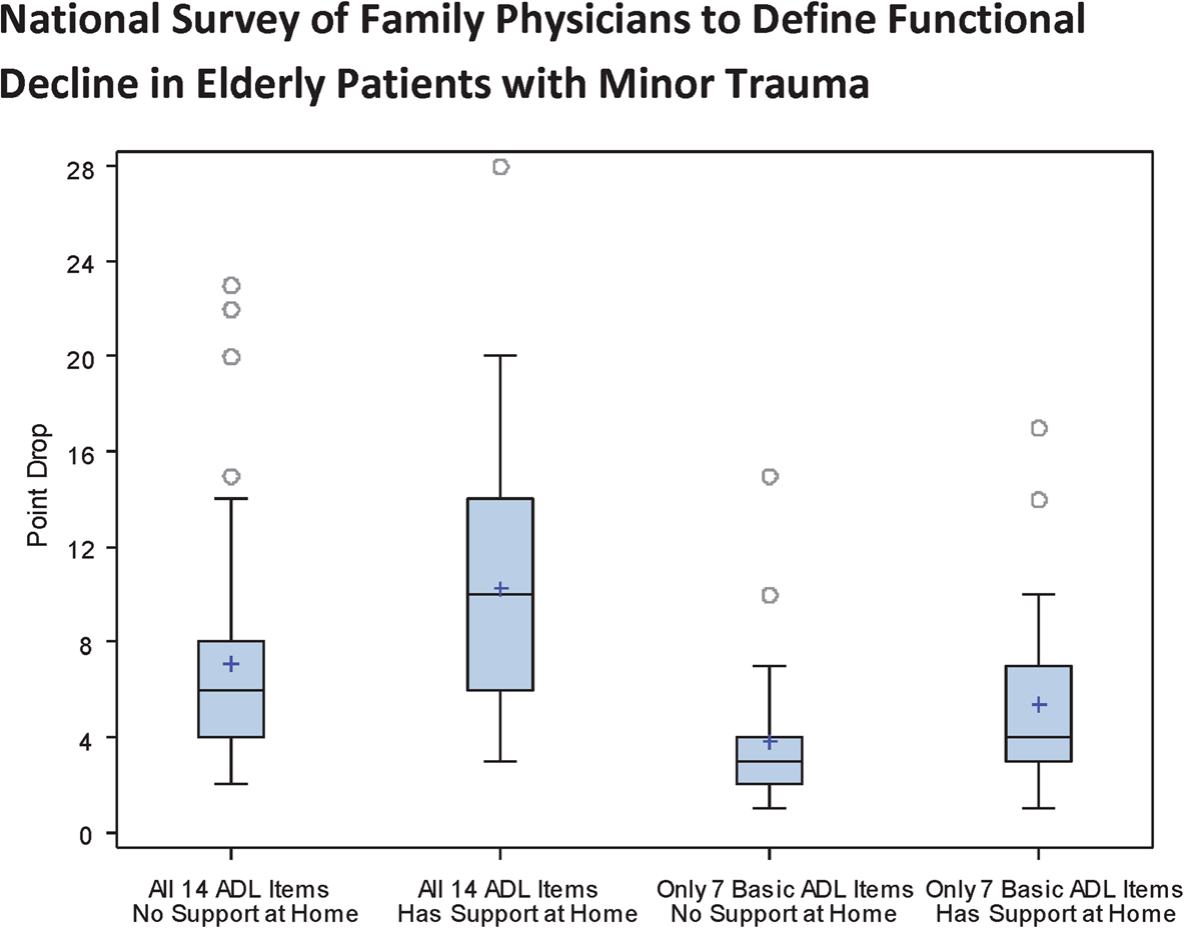
Boxplots showing distribution of family physician responses on a clinically significant point drop to define functional decline

**Fig. 2:**
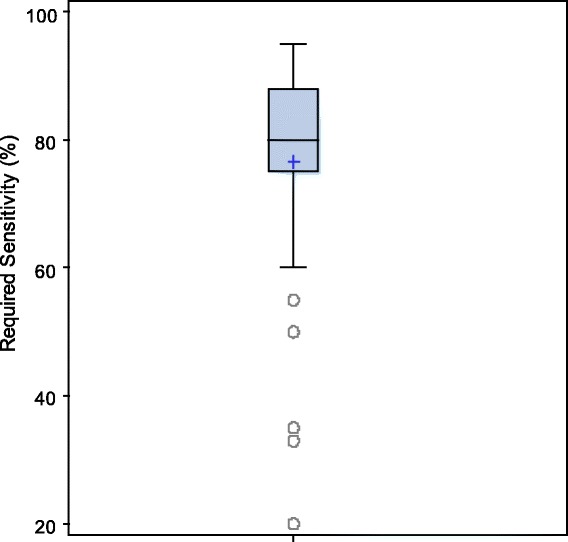
Boxplot showing distribution of family physician responses on the required sensitivity

A sensitivity of 90 % for a clinical instrument to identify elderly patients at elevated risk of subsequent functional decline would satisfy 90 % of family physicians. Physician responses on decline in function and the required sensitivity for a clinical instrument are presented in Figs. 1 and 2. As presented by the boxplots there is less variability among the family physicians in terms of the required sensitivity and the decline in function to define functional decline. There is noticeable variability among the physicians on the decline in function when it comes to all the 14 ADL items and the patient has support at home.

**Fig. 3:**
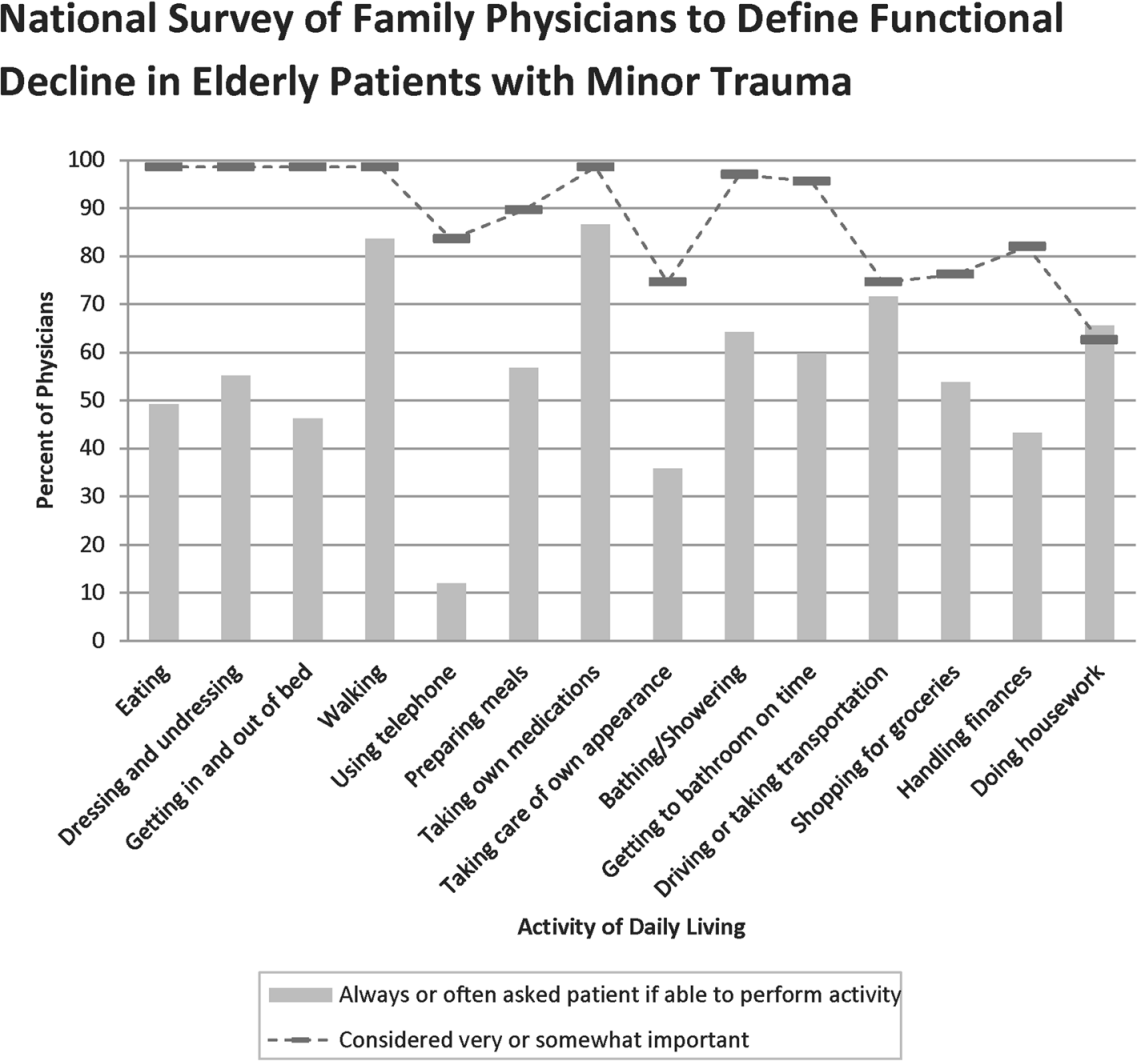
Percent of family physicians that perceive activity of daily living to be very or somewhat important in terms of performance by patients (*dashed lines*) and percent of family physicians always or often asking if patients have difficulty performing activity of daily living (*solid bars*)

Figure 3 describes family physician attitudes on assessment and importance of ADLs to functional decline. The most frequently asked ADL items by the physicians were walking, taking own medications, and driving or taking transportation. Using telephone, handling finances, and taking care of own appearance, were least frequently asked. Eating, dressing and undressing, getting in and out of bed, walking, and taking own medications were thought to be the most important items. Items thought to be less important were: ability to do housework, taking care of own appearance, and driving or taking transportation. The data showed that most of the physicians do not assess patients for most ADLs even when the ADL was considered important.

## Discussion

As the proportion of geriatric population increases and concomitant burden on healthcare system, a clinically sensible instrument to allow identification of elderly patients at an elevated risk of functional decline following a minor injury would be useful. A number of screening or assessment instruments such as the Identification of Seniors at Risk (ISAR), Hospital Admission Risk Profile (HARP), Score Hospitalier d’Evaluation du Risque de Perte d’Autonomie (SHERPA) and Triage Risk Screening Tool (TRST) exist to identify patients at risk of functional decline but they target frail individuals at imminent risk of hospitalization, institutionalisation and death and are not designed for use with independent elderly patients for a minor trauma [1, 2, 15, 16]. This study was conducted to determine necessary clinical measures, such as drop in function, needed for a new clinical decision instrument to help identify high-risk elderly patients for functional decline 6 months after experiencing a minor injury. To our knowledge, there have been no studies done on assessing the required sensitivity of an instrument to predict subsequent functional decline or what is a clinically important point drop, in function, on the OARS ADL Score.

### Point drop and sensitivity

The majority (90 %) of family physicians would consider a drop in function of 3 or more points (out of 28-points) on the OARS ADL Scale as clinically significant when all 14 basic ADL/IADL items are considered. Considering only the seven basic ADL items, 90 % of the family physicians would consider a drop in function of at least 1 point to be clinically significant. This shows the importance of the support-level received at home in identifying a clinically significant point drop to define functional decline. The physicians were asked for their opinions on the point drop in function separately based on whether the patient had support at home or not as it was suggested by our key informants that we ask the point drop questions separately based on the level of support at home.

A sensitivity of 90 % is required to pass the requirements for 90 % of family physicians for an instrument to detect previously independent patients at high risk of a functional decline at 6 months post injury. We considered the 90th percentile instead of the median or the mean since we want a solid majority of family physicians to be satisfied with the clinical measures. If we were to use the medians, only half of the physicians would be satisfied. Although the majority of family physicians had indicated a sensitivity of 90 % for a clinical instrument before using it, the results might indicate physicians’ previous habits of using highly sensitive instruments and as such asked for high sensitivity. In our study, only 50 % of the physicians had indicated a sensitivity of up to 80 %. In other words, only half of the physicians would be satisfied with a sensitivity of 80 %. The sensitivity of the commonly used Identification of Seniors at Risk (ISAR) is 81 % [23, 24]. Hence, half of the family physicians in this study require a clinical instrument greater than the ISAR.

### Relevance and assessment of ADL with respect to functional decline

Most of the ADL items were ranked as highly important to decline in function by a high proportion of family physicians but they admit that they do not routinely assess patients for all the items. One possible reason for such under-assessment is time constraints on the side of the family physicians due to a high patient to physician ratio. Another possible reason is lack of specialized training of these assessments and the instruments available for assessment. When the minor injury involves a soft tissue injury and is not apparent at the time of the visit, such as fractures and sprains, the physician is less likely to notice and take appropriate action as reported by some investigators [28]. Our results highlight the fact that although the physicians are aware of the importance of the ADL items in terms of performance by the elderly patients, they do not routinely assess for such items for various reasons.

## Conclusion

Our results show a minimal clinically important difference in function or decline in function that would satisfy 90 % of family physicians is a 3 points drop on the 28-point OARS ADL Scale. The minimal clinically important difference is 1 point when looking at the seven basic ADL items only. Our results show that ninety percent (90 %) of family physicians would accept a sensitivity of 90 % or more for any clinical instrument to identify injured seniors at elevated risk of functional decline 6 months post minor injury. Our results help distinguish a real clinical outcome for a clinical decision rule compared to what the public might perceive as an outcome.

Currently, a high percentage of family physicians do not evaluate patients for functional decline. Family physicians, however, realize and indicate the importance of such assessments. Not identifying and discharging patients at elevated risk of functional decline may lead to unwanted health outcomes including functional decline even at 6 months following a minor injury. Family physicians are encouraged to carefully assess elderly patients who sustain apparently minor trauma to ensure that early functional decline is detected and appropriate referrals are made to optimize patient outcomes. Development of a clinical instrument may help family physicians identify elderly patients at elevated risk of functional decline. The results of our study will inform the development of such a clinical decision rule. With such a clinical decision rule family physician could take the necessary and appropriate steps, including referring certain patients to a geriatric assessment clinic, to prevent functional decline from ever occurring.

## Additional file

Additional file 1: Functional Decline in Elderly Patients Questionnaire. The survey questionnaire that was administered to family physicians. (PDF 537 kb)

## Abbreviations

ADL: Activities of daily living
HARP: Hospital admission risk profile
IADL: Instrumental activities of daily living
ISAR: Identification of seniors at risk
OARS: Older Americans Resources and Services
SHERPA: Score Hospitalier d’Evaluation du Risque de Perte d’Autonomie
TRST: Triage risk screening tool
