# Supplementary material for: National survey of family physicians to define functional decline in elderly patients with minor trauma
Source: BMC Fam Pract. 2016 Aug 22;17(1):117. doi: 10.1186/s12875-016-0520-1 (PMC4994293; doi:10.1186/s12875-016-0520-1)
Supplement: Additional file 1: — Functional Decline in Elderly Patients Questionnaire. The survey questionnaire that was administered to family physicians. (PDF 537 kb) [file 12875_2016_520_MOESM1_ESM.pdf]

Questionnaire ID ___________

FUNCTIONAL DECLINE IN ELDERLY PATIENTS - FOR DR. JEFF PERRY, UNIVERSITY OF OTTAWA

Are you currently practicing family medicine AND treating patients 65 years of age and older?

☐ Yes ☐ No

If No, please return the questionnaire in the postage paid envelope

If Yes, please complete and return the questionnaire in the postage paid envelope

A. Professional Status and Practice Settings

1. Are you: ☐ Male ☐ Female

2. Year of Birth: 19_____

3. How many years have you been practicing medicine? _______ years

4. How many years of residency training have you had in total? _______ years

5. In what setting do you perform MOST of your family medicine clinical activity?

- ☐ Solo practice
- ☐ Group practice
- ☐ Hospital
- ☐ Other (specify): ____

6. On average how many patients (of any age) do you see per week? _______ # patients/week

7. On average how many patients that are 65 years of age and older do you see per week? _______ # elderly patients/week

B. Assessment for Functional Decline

8. How often do you ask elderly patients if they have difficulty performing the following activities of daily living (ADL)?

		Always	Often	Rarely	Never
a.	Eating	☐	☐	☐	☐
b.	Dressing and undressing	☐	☐	☐	☐
c.	Getting in and out of bed	☐	☐	☐	☐
d.	Walking	☐	☐	☐	☐
e.	Using telephone	☐	☐	☐	☐
f.	Preparing meals	☐	☐	☐	☐
g.	Taking own medications	☐	☐	☐	☐
h.	Taking care of own appearance	☐	☐	☐	☐
i.	Bathing/Showering	☐	☐	☐	☐
j.	Getting to bathroom on time	☐	☐	☐	☐
k.	Driving or taking transportation	☐	☐	☐	☐
l.	Shopping for groceries	☐	☐	☐	☐
m.	Handling finances	☐	☐	☐	☐
n.	Doing housework	☐	☐	☐	☐

C. Required Sensitivity

9. If a clinical decision rule were available to identify elderly patients at high-risk of functional decline 6 months after sustaining a minor injury (injury that is treated on an outpatient basis without being admitted), how sensitive would you require such a rule to be, before you would be willing to use it?

|___|___| % *(Sensitivity = probability of correctly identifying a patient at high-risk of functional decline at 6 months)*

Jeffrey J. Perry, MD, MSc, CCFP-EM *The Ottawa Hospital – Civic Campus* *(613) 798-5555 Ext. 17571 jperry@ohri.ca*

D. Measuring Functional Decline

The following 14 tasks make up the Older Americans Resources and Services (OARS) Activities of Daily Living and Instrumental Activities of Daily Living (ADL/IADL) scale:

1. Eating
2. Dressing and undressing
3. Getting in and out of bed
4. Walking
5. Using telephone
6. Preparing meals
7. Taking own medications
8. Taking care of own appearance
9. Bathing/Showering
10. Getting to bathroom on time
11. Driving or taking transportation
12. Shopping for groceries
13. Handling finances
14. Doing housework

Each task is given a point value of 0, 1 or 2 based on ability to perform the activity:

- **2 points**: can perform the activity **without help**
- **1 point**: can perform the activity but **with some help**
- **0 points**: **completely unable** to perform the activity

10. Assuming all 14 tasks are used in determining functional decline what point drop would you say is clinically significant to imply functional decline 6 months after sustaining a minor injury? *(Point drop = number of points lost due to change in ability from a baseline of being completely independent (i.e. 28 points = 14 tasks x 2 points each))*

a) Point drop if the patient has NO support at home: |___|___| point drop *(points lost from initial score of 28)*
b) Point drop if the patient has extensive support at home (i.e. lives with spouse/child who is able to help):
|___|___| point drop *(points lost from initial score of 28)*

11. If we were to only look at the basic ADL tasks (i.e. the 7 tasks listed below) what point drop would you say is clinically significant to imply functional decline 6 months after sustaining a minor injury? *(Point drop = number of points lost due to change in ability from a baseline of being completely independent (i.e. 14 points = 7 tasks x 2 points each))*

- ✓ Eating
- ✓ Dressing and undressing
- ✓ Taking care of own appearance
- ✓ Walking
- ✓ Getting in and out of bed
- ✓ Bathing/Showering
- ✓ Getting to bathroom on time

a) Point drop in basic ADL if the patient has NO support at home: |___|___| point drop *(points lost from initial score of 14)*
b) Point drop in basic ADL if the patient has extensive support at home (i.e. lives with spouse/child who is able to help):
|___|___| point drop *(points lost from initial score of 14)*

E. Relevance of Activities of Daily Living to Functional Decline

12. Six months after sustaining minor injury (injury that is treated on an outpatient basis without being admitted), how important do you consider each of the following tasks in terms of performance by an elderly patient who was completely independent prior to the injury? Assume the patient has NO support at home.

		Very Important	Somewhat Important	Less Important	Not Important
a.	Eating	☐	☐	☐	☐
b.	Dressing and undressing	☐	☐	☐	☐
c.	Getting in and out of bed	☐	☐	☐	☐
d.	Walking	☐	☐	☐	☐
e.	Using telephone	☐	☐	☐	☐
f.	Preparing meals	☐	☐	☐	☐
g.	Taking own medications	☐	☐	☐	☐
h.	Taking care of own appearance	☐	☐	☐	☐
i.	Bathing/Showering	☐	☐	☐	☐
j.	Getting to bathroom on time	☐	☐	☐	☐
k.	Driving or taking transportation	☐	☐	☐	☐
l.	Shopping for groceries	☐	☐	☐	☐
m.	Handling finances	☐	☐	☐	☐
n.	Doing housework	☐	☐	☐	☐

Thank you for taking the time to complete this questionnaire. Your input is appreciated.
